# Cytotoxic T lymphocyte effector function is independent of nucleus–centrosome dissociation

**DOI:** 10.1002/eji.201242525

**Published:** 2012-06-27

**Authors:** Winnie W Y Lui-Roberts, Jane C Stinchcombe, Alex T Ritter, Anna Akhmanova, Iakowos Karakesisoglou, Gillian M Griffiths

**Affiliations:** 1 Cambridge Institute for Medical Research, University of CambridgeCambridge, UK; 2 Department of Cell Biology, Faculty of Science, Utrecht UniversityUtrecht, The Netherlands; 3 School of Biological Sciences, University of DurhamDurham, UK

**Keywords:** Centrosome, Cytotoxic T lymphocytes, Immunological synapse, Nucleus, Polarization

## Abstract

Cytotoxic T lymphocytes (CTLs) kill tumorigenic and virally infected cells by targeted secretion of lytic granule contents. The precise point at which secretion occurs is directed by the centrosome docking at the immunological synapse (IS). The centrosome is highly dynamic in CTLs, lagging behind the nucleus in the uropod of migrating CTLs, but translocating across the entire length of the cell to dock at the IS when a target cell is recognized. While in most cell types, the centrosome is always closely associated with the nuclear membrane, in CTLs, it often appears to be dissociated from the nucleus, both in migrating cells and when forming an IS. We asked whether this dissociation is required for CTL killing, by expressing GFP-BICD2-NT-nesprin-3, which tethers the centrosome to the nucleus irreversibly. Immunofluorescence microscopy revealed that the centrosome polarized successfully to the central supramolecular activation complex (cSMAC) of the synapse in GFP-BICD2-NT-nesprin-3-expressing CTLs, with the centrosome and nucleus migrating together to the IS. CTLs in which the centrosome was “glued” to the nucleus were able to dock and release granules at the IS as effectively as mock-treated cells. These data demonstrate that CTL cytotoxicity is independent of centrosomal dissociation from the nuclear envelope.

## Introduction

Cytotoxic T lymphocytes (CTLs) are part of the adaptive immune system. They express CD8 on their cell surface, and kill virally infected and tumorigenic cells by releasing perforin and granzymes from specialized secretory lysosomes, also known as lytic granules.

When a CTL recognizes a target, an immunological synapse (IS) is formed at the interface between the two cells. The specificity of this recognition is brought about by the interaction between the T-cell receptor (TCR) on the CTL cell surface and the specific peptide presented on the MHC class I molecule that it recognizes on the target cell. The IS was described to have a “bull's eye” structure and is organized into discrete domains [1]. The central Supramolecular Activation Complex (cSMAC), which is located in the centre of the synapse, is rich in signaling molecules such as Lck, PKCθ, and TCR. Surrounding the cSMAC is the peripheral SMAC (pSMAC), an adhesion domain with integrin molecules. During the formation of the IS, there is a reorganization of actin molecules at the plasma membrane. Actin is cleared away from the centre of the synapse, and is distributed to the distal SMAC (dSMAC), the outermost ring of the synapse [2 3 4].

Secretion occurs at a very precise point in the immune synapse, next to the cSMAC and within the pSMAC. The centrosome plays a key role in directing secretion to this point. Upon TCR-mediated recognition of a target, the centrosome polarizes toward and contacts the plasma membrane adjacent to the cSMAC. The lytic granules move along microtubules and are delivered to the plasma membrane at the point determined by the centrosome [4].

**Figure 1:**
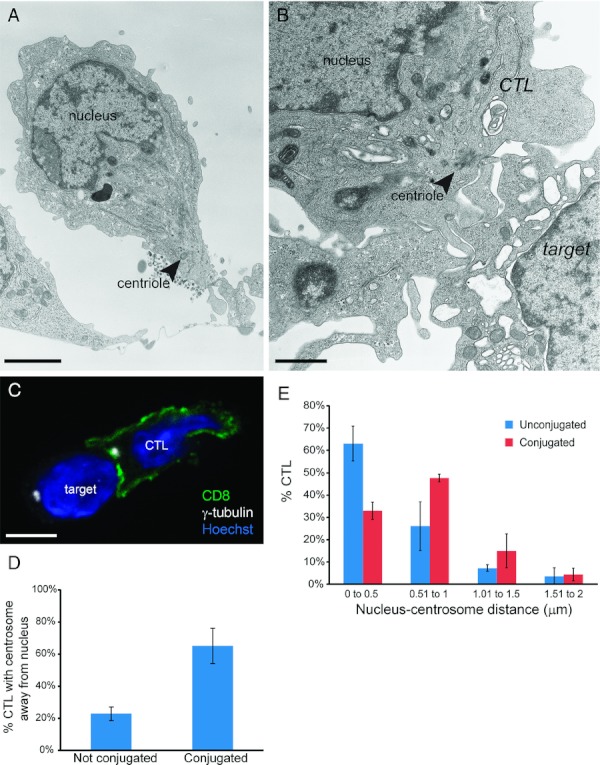
The centrosome in CTLs is often found detached from the nucleus when conjugated with target cells. (A) Electron micrograph of a migrating OT-I CTL. Bar: 2 μm. (B) Electron micrograph of an OT-I CTL conjugated to an OVA-pulsed target EL4 cell. The centriole (arrowhead), docked at the IS, is distant from the nucleus. Bar: 1 μm. (C) MeOH-fixed OT-I CTL/ target EL4 conjugates were immunolabeled with rat anti-CD8 (green) and rabbit anti-γ-tubulin (white), followed by Alexa 488-donkey anti-rat and Cy5-donkey anti-rabbit. The nuclei were stained with Hoechst (blue). A confocal slice shows a CTL centrosome docked at synapse and separated from the nucleus. Bar: 5 μm. (D) Quantitation of nuclear–centrosome separation. A total of 242 CTLs and 180 CTLs conjugated to targets were analyzed by 3D reconstruction. Number of experiments ≥ 3. Error bars = SD; *p* = 0.002, Student's *t*-test. (E) The distance between the center of γ-tubulin staining and the closest lamin B1 staining in 109 unconjugated and the same number of conjugated OT-I CTLs was measured using Bitplane Imaris software. The histogram shows the distribution of distances. Number of experiments = 3. Error bars = SD.

In CTLs, the centrosome is highly dynamic [5]. In resting cells, it is closely associated with the nucleus; while in migrating cells, it is often seen dissociated from the nucleus (Fig. 1A) and lags behind in the uropod at a varied distance. When a CTL finds a target, there is a dramatic change in the position of the centrosome, which moves from the uropod across the length of the cell to dock at the plasma membrane at the IS [4 6 7 8]. CTLs lacking the T-cell tyrosine kinase, Lck, are unable to move the centrosome to contact the plasma membrane at the synapse, and these cells are unable to kill their targets, demonstrating that centrosome docking is essential for CTL cytotoxicity [8].

The dynamic nature of the centrosome in CTLs contrasts with the close association seen between centrosome and nuclear envelope in the majority of cell types. Dissociation of the centrosome from the nuclear envelope does occur in other cell types; for example, during ciliogenesis, when it relocates to contact the plasma membrane [9]. This dissociation is required for ciliogenesis [10 11].

Centrosomal association with the nuclear membrane is mediated by nesprins, a family of type II transmembrane proteins that reside on the outer nuclear membrane. The C-terminal KASH domain of nesprins interacts with the SUN domain of SUN proteins [12], localized on the inner nuclear membrane, while the N-terminal part of nesprins can interact with different cytoskeletal proteins including microtubule-based motors kinesin-1 and dynein [13 14]. The chimeric construct GFP-BICD2-NT-nesprin-3 encodes the KASH domain of nesprin-3, which localizes to the nuclear envelope, and the N-terminal domain of Bicaudal 2, which is capable of inducing minus end-directed movement along microtubules regardless of the molecular context [15]. The expression of this construct prevents centrosome–nucleus dissociation in RanBP2-depleted U2OS cells [16].

Given the unusually dynamic nature of the centrosome in CTLs, we asked whether the centrosome needs to be dissociated from the nuclear membrane during target cell killing. We therefore made use of the GFP-BICD2-NT-nesprin-3 construct to effectively “glue” the centrosome to the nuclear membrane and asked whether CTL killing was able to proceed.

## Results

### The centrosome in CTLs is often found detached from the nucleus when conjugated with target cells

In migrating CTLs, the centrosome is found in the uropod [17 18] and often very distant from the nucleus (Fig. 1A). Similarly, when CTLs are engaged with targets, the centrosome also appears at some distance from the nucleus, as revealed by EM and immunofluorescence images of CTLs derived from OT-I TCR-transgenic mice [19] (Fig. 1B and C).

Quantification of the distance between the nuclear envelope, identified by lamin B1, the centrosome, labeled with anti-γ-tubulin, and the plasma membrane (CD8), analyzed by 3D reconstructions of confocal *z*-stacks, revealed an increase in the number of CTLs with centrosomes separated from the nucleus from 23 to 65% upon conjugation to targets (Fig. 1D). This represents a statistically significant difference of nearly threefold (*p* = 0.002, Student's *t*-test). Figure 1E shows a histogram of the distribution of distances between the CTL centrosome and the nucleus in 109 OT-I/target EL4 conjugates, as compared to the same number of unconjugated OT-I CTLs. It should be noted that the detached centrosome in unconjugated CTLs is always in the uropod, presumably of migrating cells. In contrast, when CTLs are engaged with targets, the detached centrosome is on the anterior side of the nucleus oriented toward the target at the IS.

### GFP-BICD2-NT-nesprin-3 efficiently prevents the dissociation of nucleus and centrosome in CTLs

**Figure 2:**
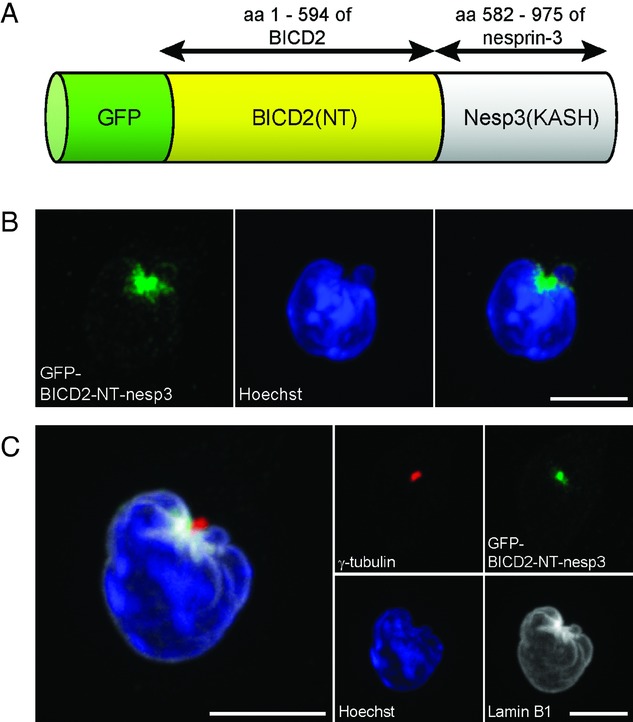
GFP-BICD2-NT-nesprin-3 efficiently prevents the dissociation of nucleus and centrosome. (A) Schematic of the GFP-BICD2-NT-nesprin-3 construct. (B) Localization of the GFP-BICD2-NT-nesprin-3 construct. OT-I CTLs transfected with GFP-BICD2-NT-nesprin-3 were fixed with PFA, immunolabeled with sheep anti-GFP, and Alexa 488-donkey anti-sheep (green). The nucleus was stained with Hoechst (blue). Bar: 5 μm. (C) The centrosome is kept in close contact with the nucleus by the expression of GFP-BICD2-NT-nesprin-3. OT-I CTLs expressing GFP-BICD2-NT-nesprin-3 were fixed with PFA, immunolabeled with mouse anti-γ-tubulin (red), sheep anti-GFP (green), rabbit anti-lamin B1 (white), followed by Cy3-donkey anti-mouse, Alexa 488-donkey anti-sheep, and Cy5-donkey anti-rabbit. The nucleus was stained with Hoescht (blue). The same cell is shown in four individual channels in the right panels, while the merge image is on the left. One hundred and ninety-eight transfected cells were analyzed by 3D reconstruction and rotation, and the centrosome of 100% of the cells was in contact with the nuclear envelope. Bar: 5 μm.

Given the frequency with which the CTL centrosome appeared distant from the nucleus upon target engagement, we asked whether the dissociation of the centrosome from the nucleus was required for CTL cytotoxicity. We therefore expressed the GFP-BICD2-NT-nesprin-3 construct (Fig. 2A) [16] in CTLs and asked whether it was possible to tether the centrosome irreversibly to the nuclear envelope in CTLs as previously described for other cell types.

When transfected into OT-I CTLs, GFP-BICD2-NT-nesprin-3 localized to the nuclear envelope, concentrated on one side of the nucleus (Fig. 2B). 3D reconstruction and analysis of *z*-stacks of 198 transfected CTLs confirmed that γ-tubulin appeared adjacent to the nuclear envelope (Fig. 2C) in 100% of transfected CTLs. It is interesting to note that lamin B1 appeared more concentrated toward the GFP-BICD2-NT-nesprin-3 construct (Fig. 2C).

### Nuclear membrane–centrosomal detachment is not required for efficient conjugate formation

In order to investigate whether nuclear membrane–centrosome dissociation was essential for efficient conjugate formation between CTLs and their targets, we used a FACS-based assay. OT-I CTLs were transfected with either GFP-BICD2-NT-nesprin-3 or a control EGFP-C1 vector, and allowed to recover for 8 h. Target EL4 cells, labeled with red PKH26 dye and pulsed with OVA peptide, were mixed with transfected CTLs for 20 min. Conjugates formed between equal numbers of green CTLs and red targets were quantitated by FACS.

**Figure 3:**
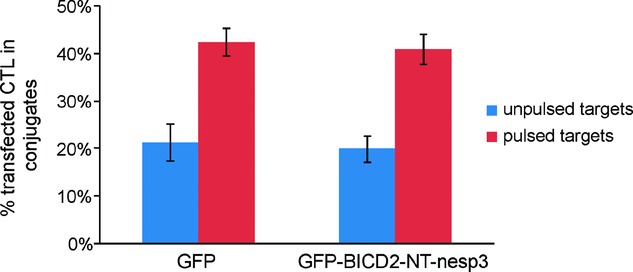
GFP-BICD2-NT-nesprin-3-transfected CTLs can form conjugates efficiently with target cells. OT-I CTLs expressing GFP or GFP-BICD2-NT-nesprin-3 were mixed with OVA-pulsed or unpulsed EL4 targets stained with the red PKH26 dye, and conjugated for 20 min and analyzed by flow cytometry. Live CTLs were gated using forward/side scatter. Flow cytometry signals positive for both the GFP channel and the red channel for PKH26 were counted as conjugates. An average of >5000 live transfected cells per condition was counted in each of six experiments. Error bars = SEM. Student's *t*-test: *p* = 0.79 for data with unpulsed EL4, *p* = 0.74 for data with pulsed EL4 between GFP and GFP-BICD2-NT-nesprin-3.

Expression of GFP-BICD2-NT-nesprin-3, compared to GFP alone, did not affect the efficiency of CTL/target conjugate formation, with 44% of CTLs forming conjugates with targets (Fig. 3). This shows that CTLs can recognize their targets and form conjugates even when the centrosome is \glued] to the nuclear membrane.

### Formation of the IS in GFP-BICD2-NT-nesprin-3-transfected CTLs

**Figure 4:**
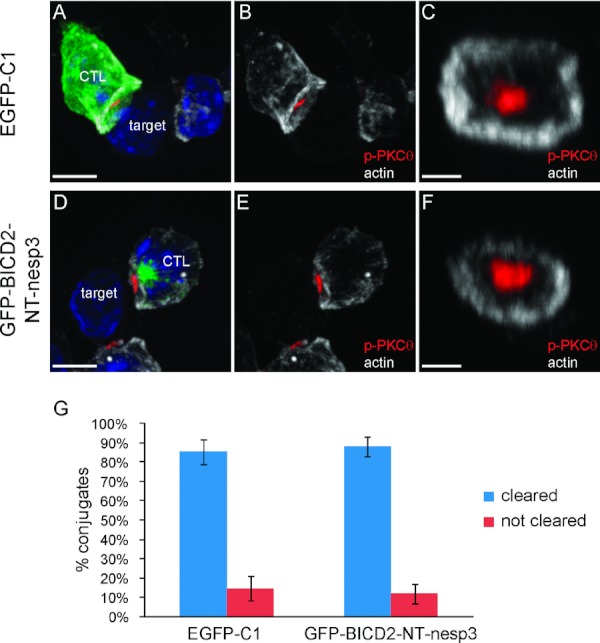
dSMAC formation in GFP-BICD2-NT-nesprin-3-transfected CTLs. (A–F) OT-I CTLs expressing (A–C) EGFP-C1 or (D–F) GFP-BICD2-NT-nesprin-3 were conjugated with OVA-pulsed EL4 targets, fixed with PFA, and processed for immunofluorescence. Mouse anti-phospho(p)-PKCθ (red), sheep anti-GFP (green), and rabbit anti-actin (white) were used, followed by Cy3-donkey anti-mouse, Alexa 488-donkey anti-sheep, and Cy5-donkey anti-rabbit. The nuclei were stained with Hoechst (blue). (A, B, D, and E) are projections of confocal *z*-stacks. Bar: 5 μm. (C and F) show the en face view of the IS. Bar: 2 μm. (G) Quantitation of actin reorganization. Phospho-PKCθ-positive conjugates were analyzed by 3D reconstruction for actin ring formation at the dSMAC region of the IS. *n* = 133 for GFP-transfected and *n* = 126 for GFP-BICD2-NT-nesprin-3-transfected CTL/target conjugates. Number of experiments = 3. Error bars = SD; *p* = 0.63, Student's *t*-test.

Next, we examined whether formation of the IS was disrupted in CTLs in which the centrosome was \glued] to the nuclear envelope. When an IS is formed, actin is reorganized to form a ring-like structure at the dSMAC of the IS [4]. Using antibodies against phospho(p)-PKCθ and actin to label the cSMAC and dSMAC, respectively, we examined the synapse in conjugates from CTLs transfected with GFP-BICD2-NT-nesprin-3. By rotating 3D reconstructions of confocal *z*-stacks, we were able to image the actin ring at the dSMAC peripheral to the cSMAC (Fig. 4). In both control CTLs and CTLs transfected with GFP-BICD2-NT-nesprin-3, actin formed a ring around the synapse, indicating the formation of dSMAC, as clearly seen in the en face views (white, Fig. 4C and F). The cSMAC, as shown by p-PKCθ localization, was in the center of the synapse. Quantitation of 126 conjugates confirmed that 88% of GFP-BICD2-NT-nesprin-3-transfected CTL/target conjugates showed definable cSMAC and dSMAC structures, as did 86% of control EGFP-C1-transfected CTLs (Fig. 4G).

### Centrosome and nuclear positioning in GFP-BICD2-NT-nesprin-3-transfected cells

Centrosome docking at the plasma membrane is critical for granule delivery and release [20]. We therefore examined the positioning of the centrosome relative to the plasma membrane in CTLs in which the centrosome was stably attached to the nuclear membrane by overexpression of GFP-BICD2-NT-nesprin-3.

**Figure 5:**
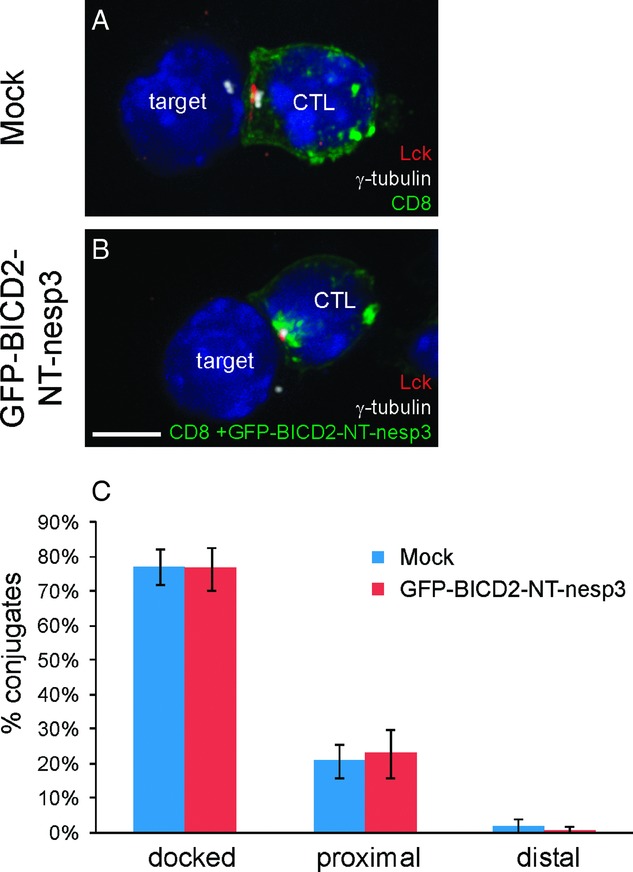
Centrosome docking at the immune synapse in GFP-BICD2-NT-nesprin-3-transfected CTLs. (A and B) GFP-BICD2-NT-nesprin-3 or mock-transfected CTLs were conjugated to OVA-pulsed EL4 targets, fixed in MeOH, and immunolabeled with mouse anti-Lck (red), rat anti-CD8 (green), sheep anti-GFP (green), and rabbit anti-γ-tubulin (white); and donkey secondary antibodies: Cy3-anti-mouse, Alexa 488-anti-rat, Alexa 488-anti-sheep, and Cy5-anti-rabbit. Nuclei were stained with Hoechst (blue). Confocal projections are shown. Bar: 5 μm. (C) Quantitation of centrosome polarization. Conjugates with a cSMAC detected by Lck were analyzed by 3D reconstruction for granule polarization. *n* = 141 for mock and *n* = 131 for GFP-BICD2-NT-nesprin-3-transfected CTL/target conjugates. Number of experiments = 3. Error bars = SD. Student's *t*-test: *p* = 0.88 for docked, 0.70 for proximal, and 0.40 for distal.

Using γ-tubulin (white) and Lck (red, Fig. 5A and B) as markers for the centrosome and the synapse, respectively, we assayed the position of the centrosome relative to the synapse using Bitplane Imaris software. The centrosome was classified as “docked”, when adjacent to the cSMAC. When the centrosome was on the synapse side of the nucleus but separated from the cSMAC, it was classified as “proximal.” Centrosomes on the distal side of the nucleus were scored as “distal.” In 76% of 131 conjugates, in which the CTLs expressed GFP-BICD2-NT-nesprin-3, the centrosome of the CTLs docked at the IS (Fig. 5C). Likewise, 77% of control CTLs showed a docked centrosome. This indicates that, when centrosome–nuclear dissociation is prevented, the centrosome can still reorientate toward the target and move to the plasma membrane.

**Figure 6:**
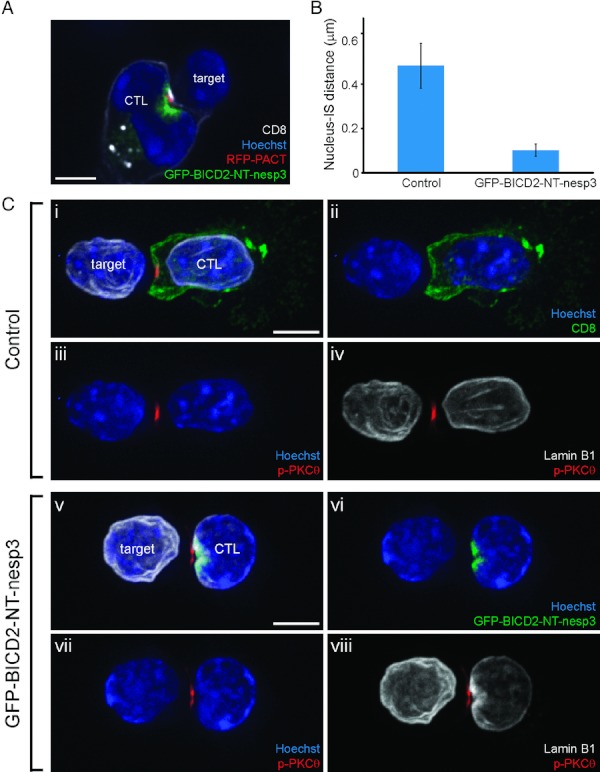
GFP-BICD2-NT-nesprin-3 brings the nucleus close to the synapse. (A) OT-I CTLs expressing GFP-BICD2-NT-nesprin-3 and RFP-PACT to mark the centrosome, were conjugated with OVA-pulsed EL4 targets, fixed with MeOH, and immunolabeled with rat anti-CD8 (white) and sheep anti-GFP (green), followed by DyLight 649-donkey anti-rat and Alexa 488-donkey anti-sheep. Nuclei were stained with Hoechst (blue). A confocal *z*-slice of a CTL with a docked centrosome at the IS is shown. Bar: 5 μm. (B) The distance between IS and nuclear envelope. OT-I CTLs were immunolabeled as is described in (C). The nearest nuclear-IS distance was measured using Bitplane Imaris software. *n* = 52 for mock-transfected and *n* = 48 for GFP-BICD2-NT-nesprin-3-transfected CTL/target conjugates. Number of experiments = 3. Error bars = SD; *p* = 0.02, Student's *t*-test. (C) CTLs transfected with GFP-BICD2-NT-nesprin-3 (v–viii) and control CTLs (i–iv) were conjugated with OVA-pulsed EL4 targets, fixed with PFA, and immunolabeled with mouse anti-phospho-PKCθ (red), rabbit anti-lamin B1 (white), and (i–iv) rat anti-CD8 (green) or (v–viii) sheep anti-GFP (green). Donkey secondary antibodies were Cy3-anti-mouse, Cy5-anti-rabbit, and Alexa 488-anti-rat (i–iv) or Alexa 488-anti-sheep (v–viii). The nuclei were stained with Hoechst (blue). Phospho-PKCθ-positive conjugates were analyzed by 3D reconstruction. Projections of *z*-stacks are shown. These images are representative of ≥48 cells. Bar: 5 μm.

What happens to the nucleus in GFP-BICD2-NT-nesprin-3-expressing CTLs when the centrosome docks? Our results showed that, when the centrosome docked at the plasma membrane in GFP-BICD2-NT-nesprin-3-transfected CTLs, the nucleus and centrosome moved together. Figure 6A shows a CTL stained with CD8, conjugated to a target, with both nuclei stained with Hoechst (blue, Fig. 6A). In this image, the centrosome, shown by expression of RFP-tagged PACT domain from pericentrin [21], was docked at the IS in a CTL expressing GFP-BICD2-NT-nesprin-3 (green). We used an antibody against lamin B1 to mark the nuclear envelope (white, Fig. 6C), and determined the nucleus–IS distance (Fig. 6C). Quantification of conjugates with cSMACs defined by pPKCθ (Fig. 6B) revealed a decrease in nucleus–IS distance from an average of 0.5 μm (SD = 0.1 μm) in control EGFP-C1-transfected CTLs (*n* = 52) to 0.1 μm (SD = 0.03 μm) in CTLs expressing GFP-BICD2-NT-nesprin-3 (*n* = 48). The difference was statistically significant (*p* = 0.02, Student's *t*-test).

These results indicate that when the centrosome is tethered to the nuclear membrane, it is still able to move to the plasma membrane and dock at the IS.

### Granule polarization is not impaired in GFP-BICD2-NT-nesprin-3-transfected cells

Lytic granule polarization toward the target and degranulation are critical steps of CTL cytotoxicity. While nucleus membrane–centrosome tethering did not impair the ability of CTLs to form an IS, we asked whether granule polarization was affected.

**Figure 7:**
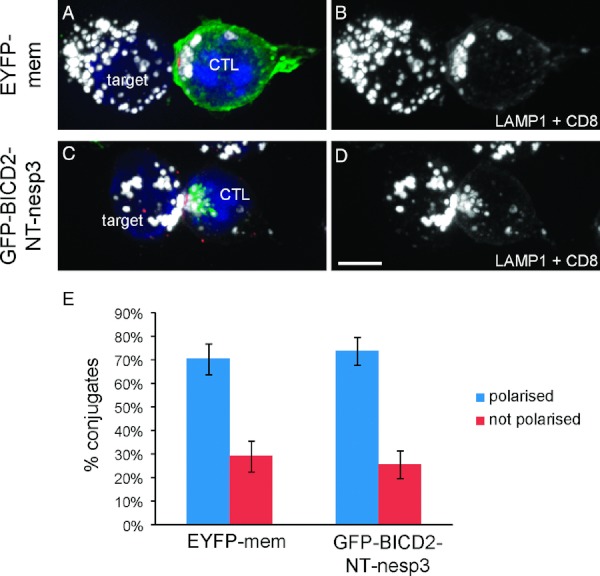
Granule polarization proceeds in GFP-BICD2-NT-nesprin-3-transfected CTLs. (A–D) OT-I CTLs transfected with (A, B) EYFP-mem or (C, D) GFP-BICD2-NT-nesprin-3 were conjugated with OVA-pulsed EL4 targets, fixed with MeOH, and immunolabeled with mouse anti-Lck (red), sheep anti-GFP (green), rat anti-CD8, followed by rat anti-LAMP1 (white), with Cy3-donkey anti-mouse, Alexa 488-donkey anti-sheep, and DyLight 649-donkey anti-rat secondary antibodies. Nuclei were stained with Hoechst (blue). Projections of confocal *z*-stacks are shown. Bar: 5 μm. (E) Quantitation of granule polarization. cSMAC formation was detected by Lck clustering analyzed by 3D reconstruction for granule polarization. *n* = 110 for EYFP-mem-transfected and *n* = 97 for GFP-BICD2-NT-nesprin-3-transfected CTL/target conjugates. Number of experiments = 3. Error bars = SD; *p* = 0.55, Student's *t*-test.

GFP-BICD2-NT-nesprin-3 or a control EYFP-mem construct that localized to the plasma membrane was transfected into OT-I CTLs. After 8–14 h, CTLs were conjugated to targets and examined by immunofluorescence using antibodies against Lck to mark the cSMAC (red, Fig. 7A and C), LAMP1 to label granules, and CD8 to show the plasma membrane of CTLs (Fig. 7). Confocal *z*-stacks of conjugates were taken, and the positions of granules relative to the IS were analyzed by 3D reconstruction. We scored a CTL as “granules polarized” when >80% of granules were clustered at the synapse (Fig. 7B and D). Figure 7E shows the results of quantitation of 110 EYFP-mem-transfected and 97 GFP-BICD2-NT-nesprin-3-transfected CTL/target conjugates, and there was no significant difference in granule polarization.

### Granule function is not impaired in GFP-BICD2-NT-nesprin-3-transfected cells

Polarized granules are not necessarily functional. This is the case for CTLs lacking Munc13-4, in which granules can dock at the IS but fail to fuse with the plasma membrane [22]. Therefore, it was important to determine whether the granules in GFP-BICD2-NT-nesprin-3-transfected cells were fully functional by testing their ability to release their contents and destroy target cells.

**Figure 8:**
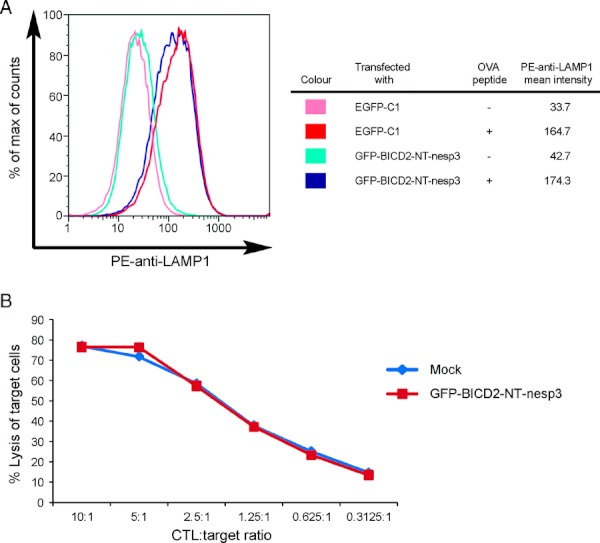
CTL effector function. (A) Representative degranulation assay (*n* = 4), in which OT-I CTLs transfected with EGFP-C1 or GFP-BICD2-NT-nesprin-3 were stimulated to exocytose with OVA peptide. GFP-positive live cells were gated for analysis of PE-anti-LAMP1 signal using flow cytometry in the presence or absence of OVA peptide. (B) Target cell death measured by the release of lactate dehydrogenase from lysed targets at various CTL:target ratios. The graph is representative of three independent experiments.

When CTL lytic granules undergo degranulation, the lysosomal membrane marker, LAMP1, appears on the plasma membrane, allowing uptake of phycoerythrin (PE)-conjugated LAMP1 antibody, which can be used to assay degranulation. CD8 CTLs are able to present peptides to each other via their own MHC class I (self-presentation), when pulsed with OVA peptide. Expressing either GFP-BICD2-NT-nesprin-3 or GFP in OT-I CTLs, we assayed degranulation after addition of OVA peptide for 2 h. We detected no significant difference in degranulation between control and GFP-BICD2-NT-nesprin-3-transfected cells (Fig. 8A). Similar results were obtained when degranulation assays were quantified at an earlier time point (30 min), and when OVA peptide was presented by target EL4 cells (data not shown).

We further investigated the killing ability of CTLs transfected with GFP-BICD2-NT-nesprin-3 using a killing assay in which target cell death was measured by release of lactate dehydrogenase (Fig. 8B). No significant difference in cytotoxicity was seen between mock-transfected and GFP-BICD2-NT-nesprin-3-transfected CTLs, consistent with the degranulation data (Fig. 8A). We therefore conclude that lytic granule exocytosis is unimpaired when nuclear membrane–centrosome dissociation is blocked.

While preventing nucleus–centrosome dissociation did not interfere with CTL cytotoxicity, we also attempted to ask whether increasing the distance between the nucleus and centrosome had an effect. Using a dominant negative SUN luminal domain construct, which has been shown to increase nucleus–centrosome distance [23], we found no difference in degranulation from CTLs transfected with a control plasmid lacking the SUN luminal domain (Supporting Information Fig. 1).

We conclude that, when the centrosome cannot detach from the nucleus during conjugate formation, it can still dock at the synapse, with the nucleus still attached. These results reveal that, centrosome docking and granule polarization to the immune synapse are independent of nucleus–centrosome dissociation.

## Discussion

The release of lytic granule contents from CTLs provides a highly effective mechanism of killing, and needs to be highly regulated. Using different mouse models and naturally occurring mutations in patients, it has been established that CTL delivery of lytic granules is a multi-step process with a number of checkpoints. When CTLs recognize target cells, signaling via the TCR triggers formation of the IS, repositioning of the centrosome toward the target, and movement of the centrosome to contact the plasma membrane where it docks within the IS [4]. The centrosome docks at the plasma membrane in response to even weak TCR signals, with a higher threshold of signaling required for granule polarization [24]. Other proteins including the AP-3 complex [25], Rab7, and RILP [26] contribute to the directional movement of granules toward the docked centrosome at the synapse. In addition, fusion of granules to the plasma membrane is controlled by the GTPase Rab27a and its effector Munc 13-4 [22 27 28 29]. Syntaxin 11 and Munc 18-2 are also required for CTL granule release [30 31 32 33].

In most cell types, the centrosome is localized close to the nuclear membrane in non-dividing cells. The KASH domain-containing nesprins provide the linker between the nuclear envelope and the cytoskeleton [23 34], and we have confirmed the expression of all four nesprins in CTLs by sequencing of RT-PCR products. Nesprins are therefore likely to play a role in nuclear–centrosomal attachment in CTLs.

Unlike the majority of cell types, the centrosome is highly dynamic in CTLs and adopts different positions depending on whether the cells are migrating or interacting with targets. Centrosome docking at the synapse is necessary for lytic granule secretion. In CTLs lacking Lck, the centrosome can reposition toward the synapse, but cannot dock at the plasma membrane, and granule release is abolished [8]. These and other studies [4] showed that, when the centrosome is docked at the synapse, it is often separated from the nuclear envelope. In this study, we found centrosome–nuclear envelope separation in 65% of CTLs conjugated to targets — a significantly higher number than in CTLs without targets. We therefore asked whether centrosomal separation from the nuclear envelope is required for docking at the plasma membrane and release of lytic granules at the IS. Our data show that, even when the centrosome is irreversibly glued to the nuclear membrane, it is able to dock at the plasma membrane and lytic granules are secreted at the IS.

Why then is the centrosome so dynamic in CTLs? One possibility is that centrosomal movement is required for movement of the cells themselves. CTLs migrate in an amoeboid fashion, with a leading edge, the cell body that contains the nucleus, and a lagging tail known as the uropod (for review, see [35]). During migration, the centrosome lags behind the nucleus, often at a distance. Preliminary results suggest that GFP-BICD2-NT-nesprin-3-transfected CTLs migrate more slowly than control transfected CTLs. However, our results suggest that migration is unlikely to be inhibited significantly, as target cell killing is unimpaired. Further studies are required to address the role of centrosomal detachment in migration of CTLs.

## Materials and methods

### Antibodies

Primary antibodies used for immunofluorescence were: rabbit anti-γ-tubulin (Sigma-Aldrich), anti-lamin B1 (Abcam), and anti-actin (Sigma-Aldrich); mouse anti-γ-tubulin (clone TU-30, Abcam), anti-Lck (clone 3a5, Millipore), and anti-pT538-PKCθ (clone 19/PKC, BD Biosciences); sheep anti-GFP (AbD Serotec); rat anti-CD8 (clone YTS192, H. Waldmann, University of Oxford), and anti-LAMP1 (clone 1D4B, Developmental Studies Hybridoma Bank). Alexa Fluor-conjugated secondary antibodies were purchased from Invitrogen; DyLight-, Cy3-, and Cy5-conjugated secondary antibodies were obtained from Jackson ImmunoResearch. For degranulation assays, PE-conjugated rat anti-LAMP1 (clone 1D4B, eBioscience) was used.

### Cell culture

Spleens isolated from OT-I mice were disrupted through 70 μm cell strainers (BD Biosciences), washed once and, for stimulation, resuspended in CTL medium containing Iscove's Modified Dulbecco's Media (Invitrogen), 10% FCS, 2 mM L-glutamine, 50 μM β-mercaptoethanol, 50 U/mL penicillin and streptomycin, 100 U/mL Proleukin IL-2 (Novartis), and 10 nM OVA_257-264_ peptide [SIINFEKL] (GenScript). OT-I CTLs were used for experiments between 6 and 9 days after stimulation. H-2K^b^ EL4 target cells were maintained in DMEM, 10% FCS, 2 mM L-glutamine.

### Constructs and transfection

EGFP-C1 and EYFP-mem were obtained from Clontech. GFP-BICD2-NT-nesprin-3, which contains amino acids 1–594 of BICD2 and amino acids 582–975 of nesprin-3 [16], as well as SP-GFP and SP-GFP-DN-SUNL [23] were described previously. RFP-PACT was a kind gift of S. Munro [21]. For transfections, the Amaxa mouse T-cell transfection kit (Lonza) was used. Typically, 5 × 10^6^ cells were transfected with 3 μg of DNA using the X-001 program on the Amaxa Nucleofector II machine (Lonza).

### Immunofluorescence and electron microscopy

Cells were rinsed with warmed serum-free medium, and plated onto multiwell slides (C.A. Hendley, Essex, UK) 15 min before fixation with MeOH for 5 min at −20°C; alternatively, to allow better preservation of nuclear structure, fixed with 3% PFA, quenched with 50 mM NH_4_Cl in PBS, followed by permeabilization with cold MeOH. After blocking in PGA (PBS, 0.2% gelatin, 0.02% NaN_3_), the cells were incubated with primary antibodies for 45 min, washed in PGA, followed by a 45-min incubation with fluorophore-conjugated secondary antibodies. The slides were washed in PGA, mounted with a glycerol-based mounting medium.

Slides were examined at ambient temperature through a 100× oil immersion lens (NA 1.4) on the Andor Revolution spinning disk confocal system. Andor iQ was the image acquisition program used; 3D analysis of *z*-stacks was carried out using Bitplane Imaris 7.2 software; Adobe Photoshop CS3 and Illustrator 10 were used to generate figures from digital images.

For immunofluorescence of CTL/target conjugates, EL4 target cells were pulsed with 1 μM OVA peptide in normal EL4 culture medium for 1 h at 37°C, with gentle shaking every 20 min. The pulsed EL4 was washed twice with normal medium. OT-I CTLs and EL4 cells were then independently rinsed once and resuspended in serum-free medium at a density of 1–1.5 × 10^6^ cells/mL. CTLs and target cells were mixed at 1:1 ratio, and incubated for 20–25 min at 37°C on multiwell slides before fixation.

Electron microscopy was carried out as described in [24].

### Conjugation assay

OT-I CTLs were transfected with GFP or GFP-BICD2-NT-nesprin-3, and allowed to recover at 37°C for 8 h. Target EL4 cells were incubated in the absence or presence of the OVA peptide for 1 h at 37°C, rinsed once in serum-free medium, and stained with the PKH26 dye using PKH26 Red Fluorescent Cell Linker Kit (Sigma-Aldrich). The cells were stained for 2 min at room temperature, with gentle mixing using a pipette; then quenched with 1 mL of FCS for 1 min, and washed four times in complete medium. Transfected CTLs and EL4 cells were mixed at 1:1 ratio and allowed to conjugate at 37°C for 20 min. The conjugates were transferred to ice and cooled with 3 mL of cold FACS buffer (0.2% BSA in PBS) to stop movements, before pelleted at 4°C. The pellets were resuspended very gently with wide-bore pipette tips just before being analyzed on a FACSCalibur flow cytometer (Becton Dickinson). Live transfected CTLs were gated using forward/side scatter. Data were analyzed using the FlowJo software. Double positives for the FL1 GFP channel and the FL2 red channel were counted as conjugates. The efficiency of conjugate formation was calculated as the percentage of red and green doublets as a percentage of the total number of transfected CTLs.

### Degranulation assay

OT-I CTLs were transfected with EGFP-C1 or GFP-BICD2-NT-nesprin-3, and incubated for 8 h at 37°C. CTLs and EL4 targets were mixed at a ratio of 1:1, and incubated for 2 h at 37°C in the presence of PE-conjugated anti-LAMP1. Cells were then cooled in ice-cold PBS and pelleted at 4°C, before being resuspended in ice-cold FACS buffer. The samples were analyzed using a FACSCalibur flow cytometer (Becton Dickinson). Live transfected CTLs were gated using forward/side scatter and FL1 channel for the GFP fluorescence signal. Data were analyzed using the FlowJo software.

### Cytotoxicity assay

OT-I CTLs were transfected in the absence or presence of GFP-BICD2-NT-nesprin-3, and incubated for 6–7 h at 37°C. Cytotoxicity assays were carried out as described previously using the Promega CytoTox 96 Non-radioactive Cytotoxicity Assay [24]. In short, CTLs and OVA-peptide-pulsed EL4 target cells were mixed in various titration ratios in round-bottom 96-well plates, and incubated for 4 h at 37°C. The release of lactate dehydrogenase from the lysed target cells causes a colorimetric change of the assay substrate, which can be detected by a spectrophotometer at 490 nm.

## Abbreviations

cSMAC: central supramolecular activation complex
dSMAC: distal SMAC
IS: immunological synapse
pSMAC: peripheral SMAC

## Notes

Disclaimer: Supplementary materials have been peer-reviewed but not copyedited.

Figure 1 Increasing nucleus-centrosome distance does not affect CTL degranulation. OT-I CTLs transfected with SP-GFP or SP-GFP-DN-SUNL were stimulated to exocytose with the OVA peptide. GFP-positive live cells were gated for analysis of PE-anti-LAMP1 signal by flow cytometry. The graph shows a typical degranulation assay. There was an increase in mean fluorescence intensity for PE-anti-LAMP1 upon the addition of the OVA peptide (table), but there was no significant difference between the SP-GFP-DN-SUNL-transfected and control CTLs.
