# Supplementary material for: Cytotoxic T lymphocyte effector function is independent of nucleus–centrosome dissociation
Source: Eur J Immunol. 2012 Jun 27;42(8):2132–41. doi: 10.1002/eji.201242525 (PMC3470926; doi:10.1002/eji.201242525)
Supplement: Figure 1 — Increasing nucleus-centrosome distance does not affect CTL degranulation. OT-I CTLs transfected with SP-GFP or SP-GFP-DN-SUNL were stimulated to exocytose with the OVA peptide. GFP-positive live cells were gated for analysis of PE-anti-LAMP1 signal by flow cytometry. The graph shows a typical degranulation assay. There was an increase in mean fluorescence intensity for PE-anti-LAMP1 upon the addition of the OVA peptide (table), but there was no significant difference between the SP-GFP-DN-SUNL-transfected and control CTLs. [file eji0042-2132-SD1.pdf]

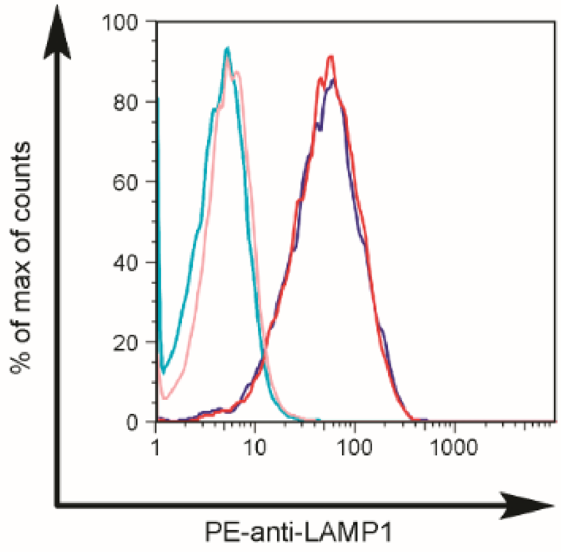

Colour	Transfected with	OVA peptide	PE-anti-LAMP1 mean intensity
	SP-GFP	-	6.07
	SP-GFP	+	66.18
	SP-GFP-DN-SUNL	-	5.15
	SP-GFP-DN-SUNL	+	64.66

Supporting information

Figure 1: Increasing nucleus-centrosome distance does not affect CTL degranulation.

OT-I CTLs transfected with SP-GFP or SP-GFP-DN-SUNL were stimulated to exocytose with the OVA peptide. GFP-positive live cells were gated for analysis of PE-anti-LAMP1 signal by flow cytometry. The graph shows a typical degranulation assay. There was an increase in mean fluorescence intensity for PE-anti-LAMP1 upon the addition of the OVA peptide (table), but there was no significant difference between the SP-GFP-DN-SUNL-transfected and control CTLs.
